# Supplementary material for: Dynamic X-ray imaging with screen-printed perovskite CMOS array
Source: Nat Commun. 2024 Feb 21;15:1588. doi: 10.1038/s41467-024-45871-2 (PMC10881555; doi:10.1038/s41467-024-45871-2)
Supplement: Supplementary file 1 — Supplementary Information [file 41467_2024_45871_MOESM1_ESM.pdf]

# Supplementary Information

# Dynamic X-ray imaging with screen-printed perovskite CMOS array

Yanliang Liu[1,7], Chaosong Gao[2,7], Dong Li[1], Xin Zhang[3], Jiongtao Zhu[3], Meng Wu[2], Wenjun Liu[1], Tongyu Shi[1], Xingchen He[1], Jiahong Wang[1], Hao Huang[1], Zonghai Sheng[4,5], Dong Liang[3,4,5], Xue-Feng Yu[1,5*], Hairong Zheng[4,5,6*], Xiangming Sun[2*], Yongshuai Ge[3,4,5,6*]

[1] Materials Interfaces Center, Shenzhen Institute of Advanced Technology, Chinese Academy of Sciences, Shenzhen, Guangdong, 518055, China

[2] Key Laboratory of Quark and Lepton Physics, Central China Normal University, Wuhan, Hubei 430079, China

[3] Research Center for Medical Artificial Intelligence, Shenzhen Institute of Advanced Technology, Chinese Academy of Sciences, Shenzhen, Guangdong, 518055, China

[4] Paul C Lauterbur Research Center for Biomedical Imaging, Shenzhen Institute of Advanced Technology, Chinese Academy of Sciences, Shenzhen, Guangdong, 518055, China

[5] Key Laboratory of Biomedical Imaging Science and System, Chinese Academy of Sciences, Shenzhen, Guangdong, 518055, China

[6] National Innovation Center for Advanced Medical Devices, Shenzhen, Guangdong, 518131, China

[7] These authors contributed equally: Yanliang Liu, Chaosong Gao.

*(*Corresponding authors: xf.yu@siat.ac.cn, hr.zheng@siat.ac.cn, xmsun@phy.ccnu.edu.cn, ys.ge@siat.ac.cn)*

## The SEM images of the CMOS X-ray detector

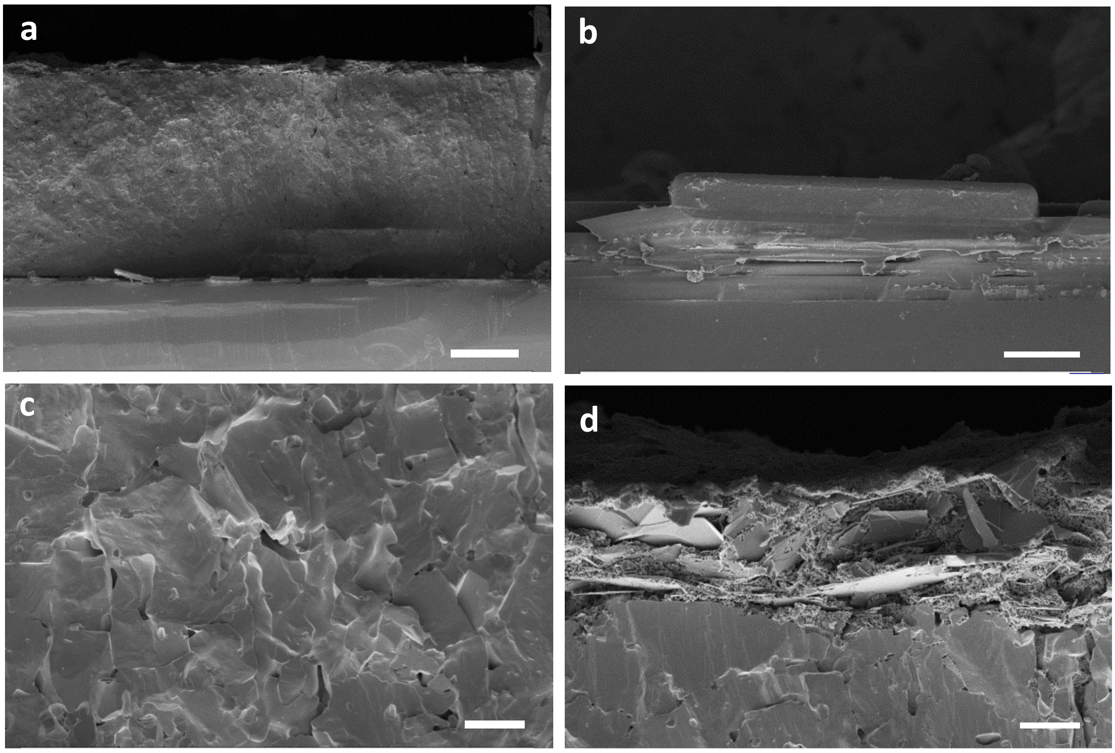


**Supplementary Fig. 1 | SEM images of the CMOS based perovskite X-ray detector. a,** overall cross-section of the CMOS X-ray detector with 300 µm thick $CsPbBr_3$. The scale bar denotes 100 µm. **b,** the bottom CMOS array. The scale bar denotes 8 µm. **c,** internal of $CsPbBr_3$ thick film. The scale bar denotes 6 µm. **d,** the top carbon electrode with thickness of about 18 µm. The scale bar denotes 6 µm. As seen, the screen-printed $CsPbBr_3$ thick film has a dense morphology and exhibits good affinity with the CMOS array.

## The CMOS array

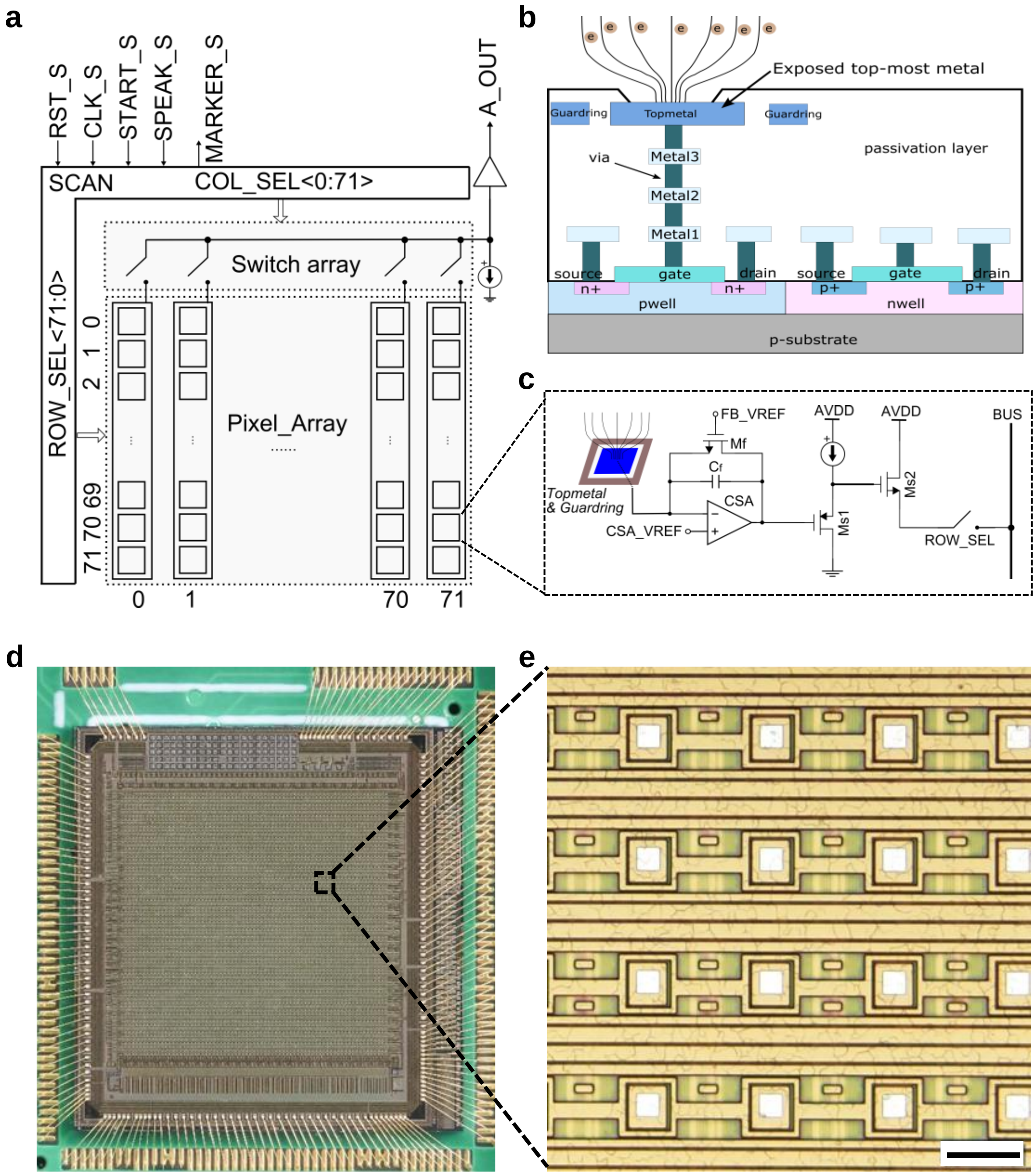


**Supplementary Fig. 2 | Details of the CMOS array. a,** diagram of the CMOS array, including a pixel array, a switch array, a scan module and an analog buffer. **b,** illustration of the cross section of the CMOS array. **c,** individual pixel containing a charge collection electrode (Topmetal), charge sensitive amplifier (CSA) and a two-stage source follower. Specifically, CSA_VREF denotes for CSA reference voltage, FB_VREF denotes for feedback transistor gate voltage, Cf denotes for feedback capacitor, Mf denotes for feedback transistor, AVDD denotes for analog power supply, Ms1 denotes for the input transistor of the first-stage source follower, Ms2 denotes for the input transistor of the second-stage source follower, ROW_SEL denotes for row selection, BUS denotes for signal bus. **d,** microphotograph of the CMOS array (20×). **e,** microphotograph of the CMOS pixels (200×). The scale bar denotes 50 μm.

## The gate voltage altered pixel response

For the designed CMOS, the dark current ($I_d$) is assumed to be fixed under a certain external electric field. Moreover, the output baseline voltage $V_o$, which is expressed as

$$V_o = I_d \times \frac{1}{\mu_n C_{ox} \frac{W}{L}(V_G - V_S - V_{th})}, \quad (1)$$

where $I_d$ denotes the dark current, $\mu_n$ denotes the electron mobility, $C_{\mathrm{ox}}$ denotes the gate oxide capacitance per unit area, $W$ and $L$ denote the width and length of the feedback transistor, respectively, $V_{\mathrm{G}}$ denotes the gate voltage of the feedback transistor, $V_{\mathrm{S}}$ and $V_{\mathrm{th}}$ denote the source voltage and the threshold voltage of the feedback transistor, respectively, both of which are fixed in the front-end circuit. Therefore, the output baseline voltage $V_{\mathrm{o}}$, corresponds to Analog to Digital Converters (ADC) value, decreases as the gate voltage $V_{\mathrm{G}}$ increases.

In particular, a Charge Sensitive Amplifier (CSA) with a suppressing dark current circuit used in the CMOS array is shown below. The dark current from the detector is absorbed by a transistor. The current generated by the transistor is automatically adjusted according to the feedback circuit which is composed of a resistor, a capacitor and an open-loop amplifier to match the dark current of the detector. Hence, the baseline of the output node can be obtained with the same level of the reference voltage node. As a consequence, the output dynamic range of the CSA is not affected by the dark current.

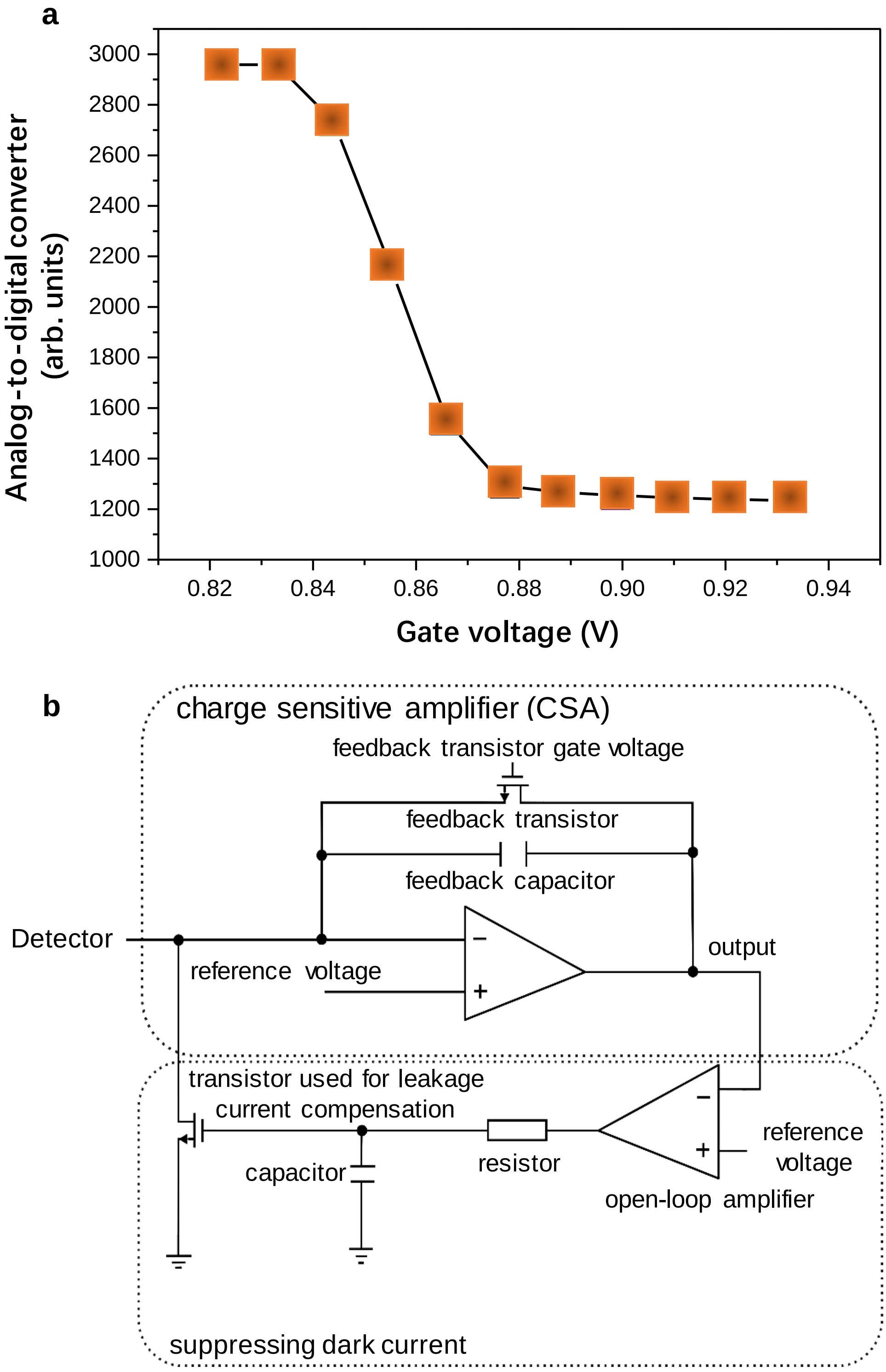


**Supplementary Fig. 3 | The gate voltage-controlled pixel analog-to-digital converter response. a,** certain gate voltages are calibrated in prior and adopted to neutralize the leakage current in every pixel to avoid saturation. **b,** a charge sensitive amplifier with a suppressing dark current circuit used in the CMOS array.

## The viscosity of the $CsPbBr_3$ precursor paste

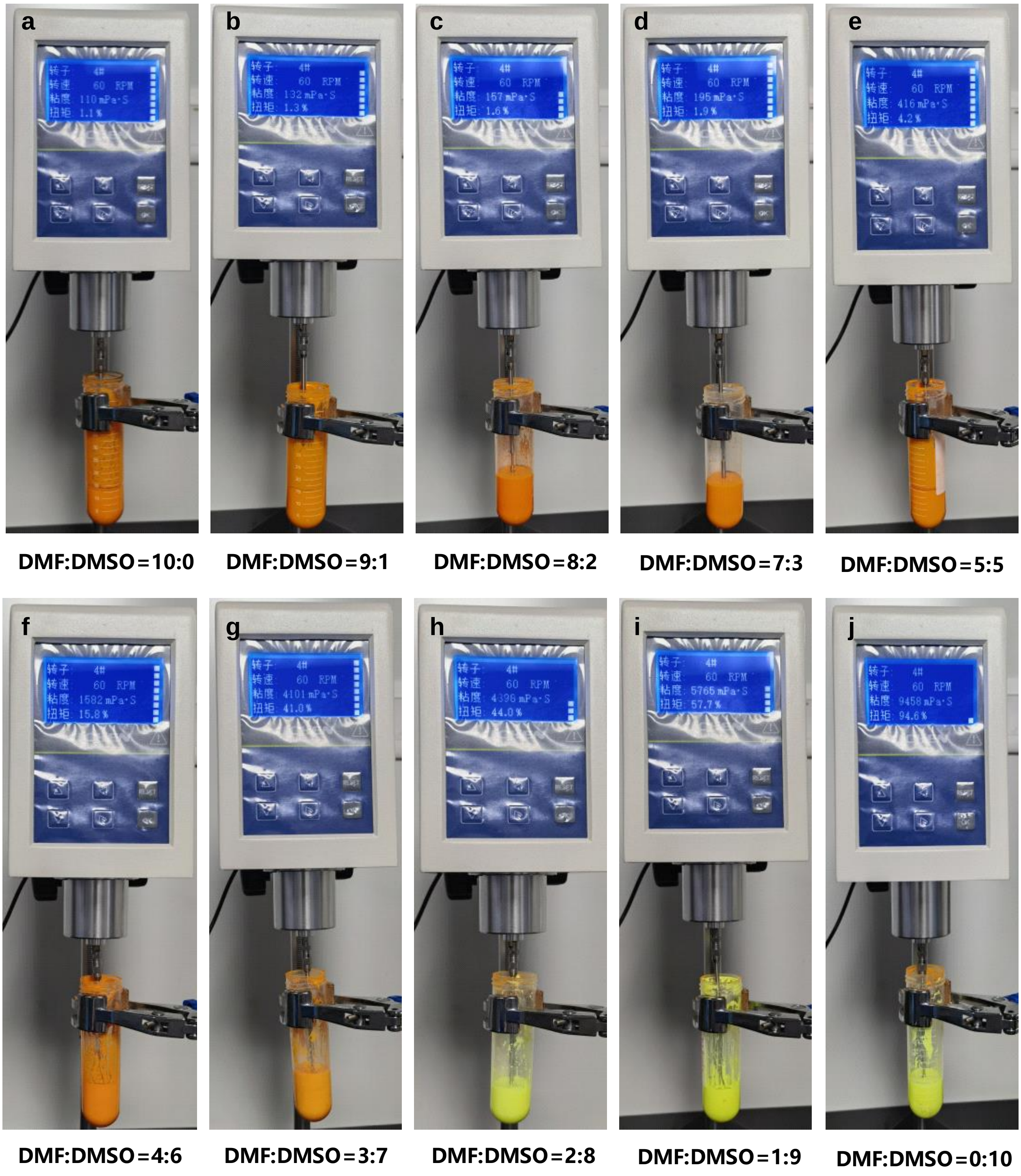


**Supplementary Fig. 4 | The viscosity of the $CsPbBr_3$ precursor paste.** The Dimethylformamide (DMF) / Dimethyl sulfoxide (DMSO) ratios are: **a**, 10:0. **b**, 9:1. **c**, 8:2. **d**, 7:3. **e**, 5:5. **f**, 4:6. **g**,3:7. **h**, 2:8. **i**, 1:9. **j**, 0:10. The viscosity of the $CsPbBr_3$ precursor paste gradually grows from 110 mPa·s to 9458 mPa·s as the dimethyl sulfoxide content in mixed solvent increases without any solid additive doping.

# The component of the $CsPbBr_3$ precursor paste

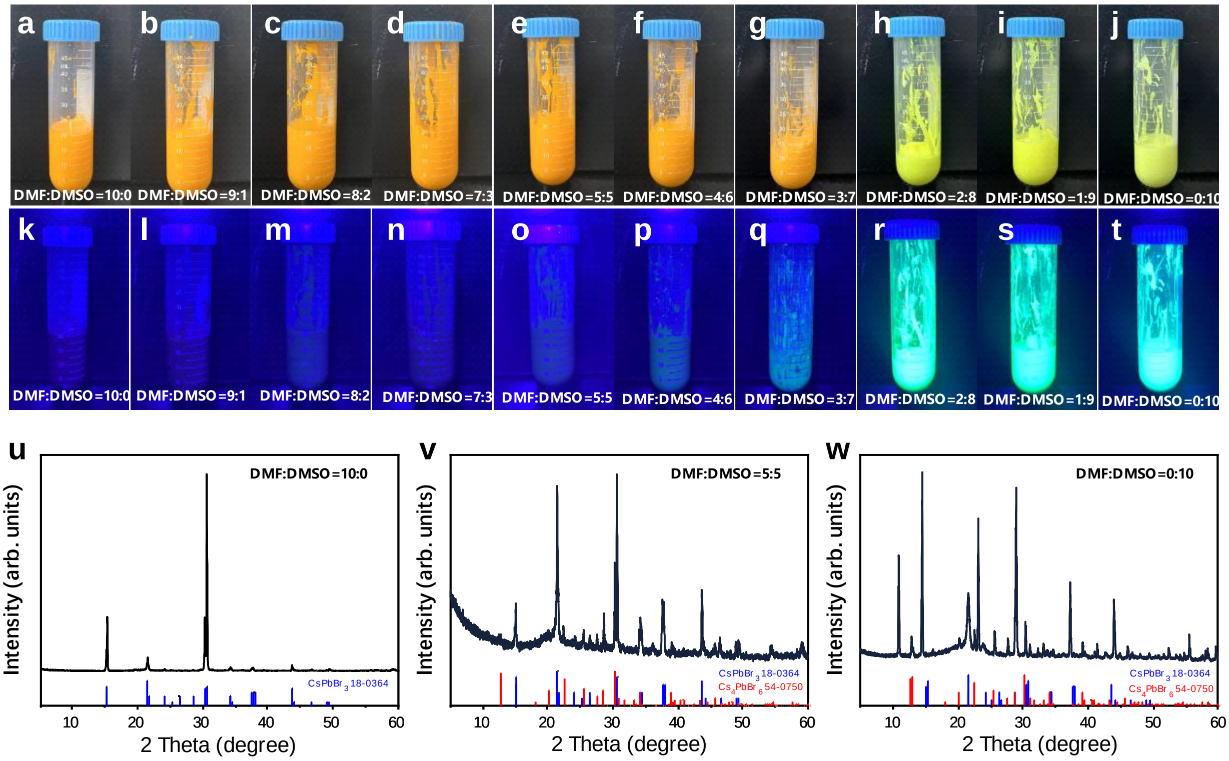


**Supplementary Fig. 5 | Component of the $CsPbBr_3$ thick film through screen-printing.** The Dimethylformamide (DMF) / Dimethyl sulfoxide (DMSO) ratios are: **a**, 10:0. **b**, 9:1. **c**, 8:2. **d**, 7:3. **e**, 5:5. **f**, 4:6. **g**,3:7. **h**, 2:8. **i**, 1:9. **j**, 0:10. Moreover, the Dimethylformamide (DMF) / Dimethyl sulfoxide (DMSO) ratios are: **k**, 10:0. **l**, 9:1. **m**, 8:2. **n**, 7:3. **o**, 5:5. **p**, 4:6. **q**,3:7. **r**, 2:8. **s**, 1:9. **t**, 0:10. The initial perovskite paste with Dimethylformamide (DMF) solvent is in yellow color and has pure 3D $CsPbBr_3$. As the Dimethyl sulfoxide (DMSO) solvent increases, the color slowly changes from yellow to green. During such transition, multiple material phases including $CsPbBr_3$ and $Cs_4PbBr_6$ are formed. The results in **a-j** are generated with white light illumination, and the results in **k-t** are generated with illumination of 365 nm light. **u**, the X-ray diffraction analysis (XRD) spectra of the $CsPbBr_3$ films with DMF/DMSO=10:0. **v,** the XRD spectra of the $CsPbBr_3$ films with DMF/DMSO=5:5. **w,** the XRD spectra of the $CsPbBr_3$ films with DMF/DMSO=0:10.

## The phase transition of $CsPbBr_3$

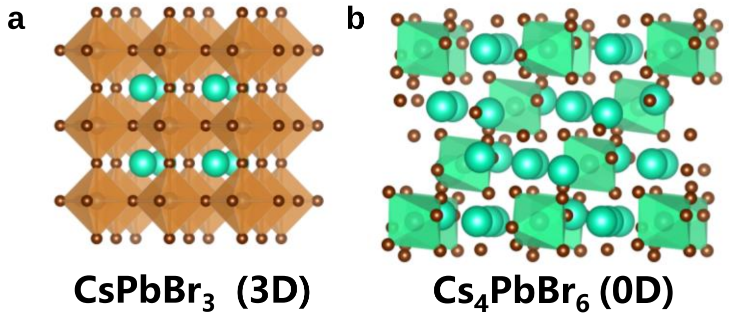


c

$$CsBr + PbBr_2 \rightarrow CsPbBr_3$$

$$4CsBr + PbBr_2 \rightarrow Cs_4PbBr_6$$

$$CsPbBr_3 + 3CsBr \rightarrow Cs_4PbBr_6$$

$$Cs_4PbBr_6 + 3PbBr_2 \rightarrow 4CsPbBr_3$$

**Supplementary Fig. 6 | The phase transition of $CsPbBr_3$. a**, illustration of $CsPbBr_3$ structure. **b**, illustration of $Cs_4PbBr_6$ structure. **c**, in $CsPbBr_3$ precursor paste, equal molar amounts of CsBr and $PbBr_2$ are used to form pure 3D $CsPbBr_3$. The high solubility of $PbBr_2$ in Dimethyl sulfoxide (DMSO) relative to CsBr leads to a phase conversion from $CsPbBr_3$ to CsBr-rich $Cs_4PbBr_6$ with moles ratio of $CsBr/PbBr_2 = 4/1$. During the heating procedure (DMF/DMSO solvents are evaporated), the dissolved CsBr and $PbBr_2$ precursors are precipitated out. Eventually, the $Cs_4PbBr_6$ turns back to $CsPbBr_3$.

## The morphology of $CsPbBr_3$ thick films

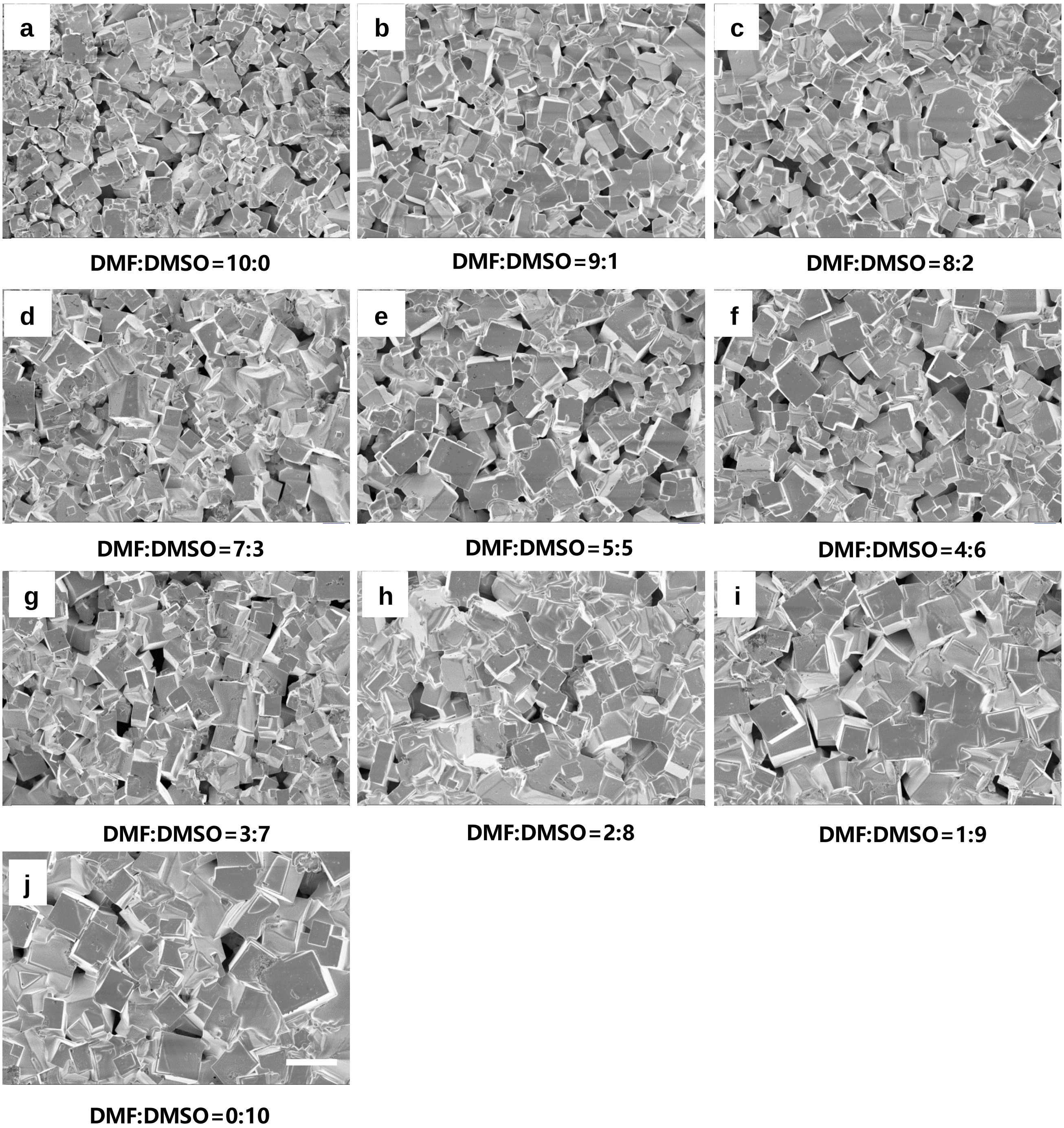


**Supplementary Fig. 7 | Images of $CsPbBr_3$ thick films with various DMF/DMSO mixed solvent.** The Dimethylformamide (DMF) / Dimethyl sulfoxide (DMSO) ratios are: **a**, 10:0. **b**, 9:1. **c**, 8:2. **d**, 7:3. **e**, 5:5. **f**, 4:6. **g**,3:7. **h**, 2:8. **i**, 1:9. **j**, 0:10. As seen, the crystal size inside the $CsPbBr_3$ thick films grows as the DMSO content increases. The scale bar denotes 20 $\mu m$ and is applied for all panels in this figure.

## The screen-printed $CsPbBr_3$ films with various patterns and sizes

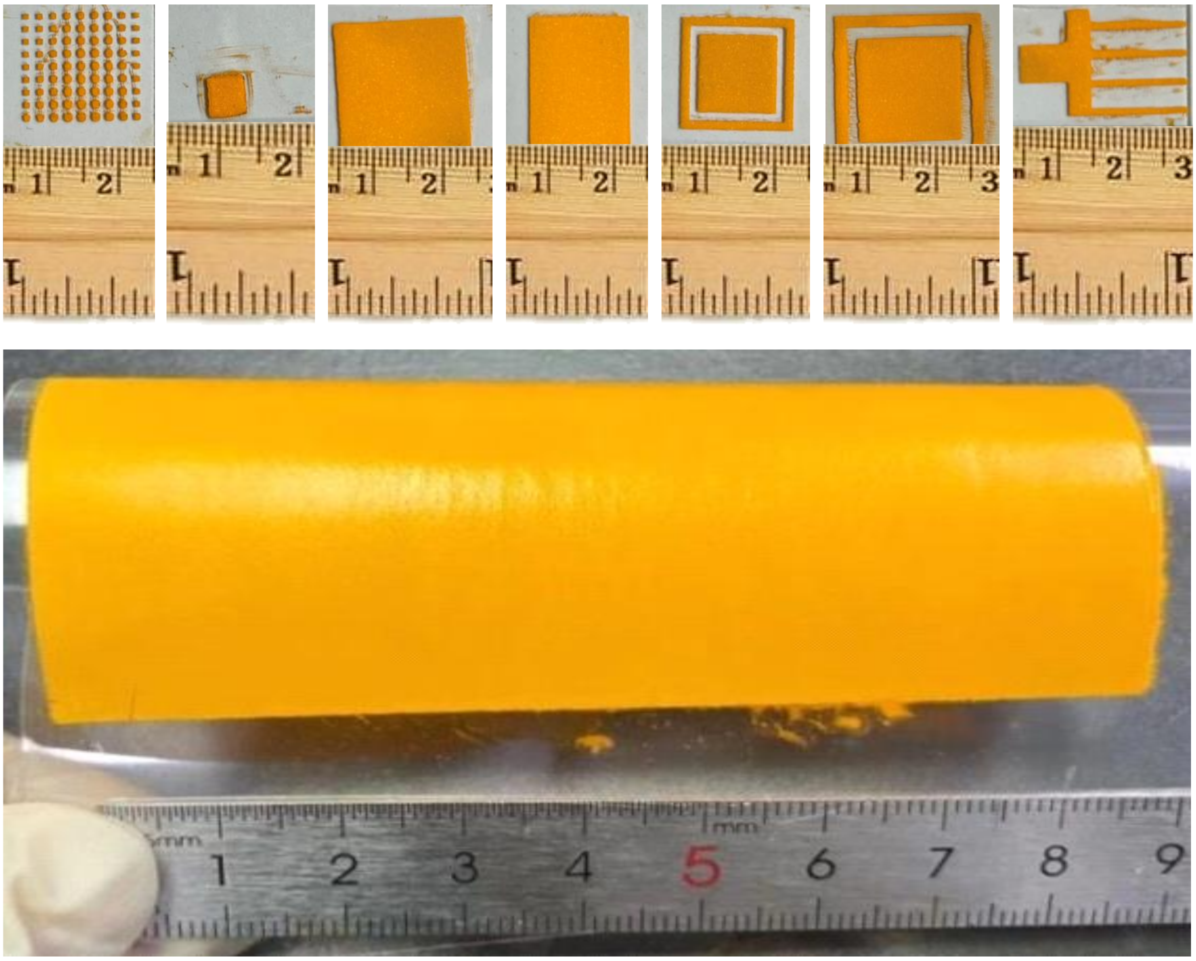

**Supplementary Fig. 8 | The screen-printed $CsPbBr_3$ films with various sizes and patterns.** The screen-printing technique is able to print various thick patterns from $CsPbBr_3$.

## The thick screen-printed $CsPbBr_3$ films

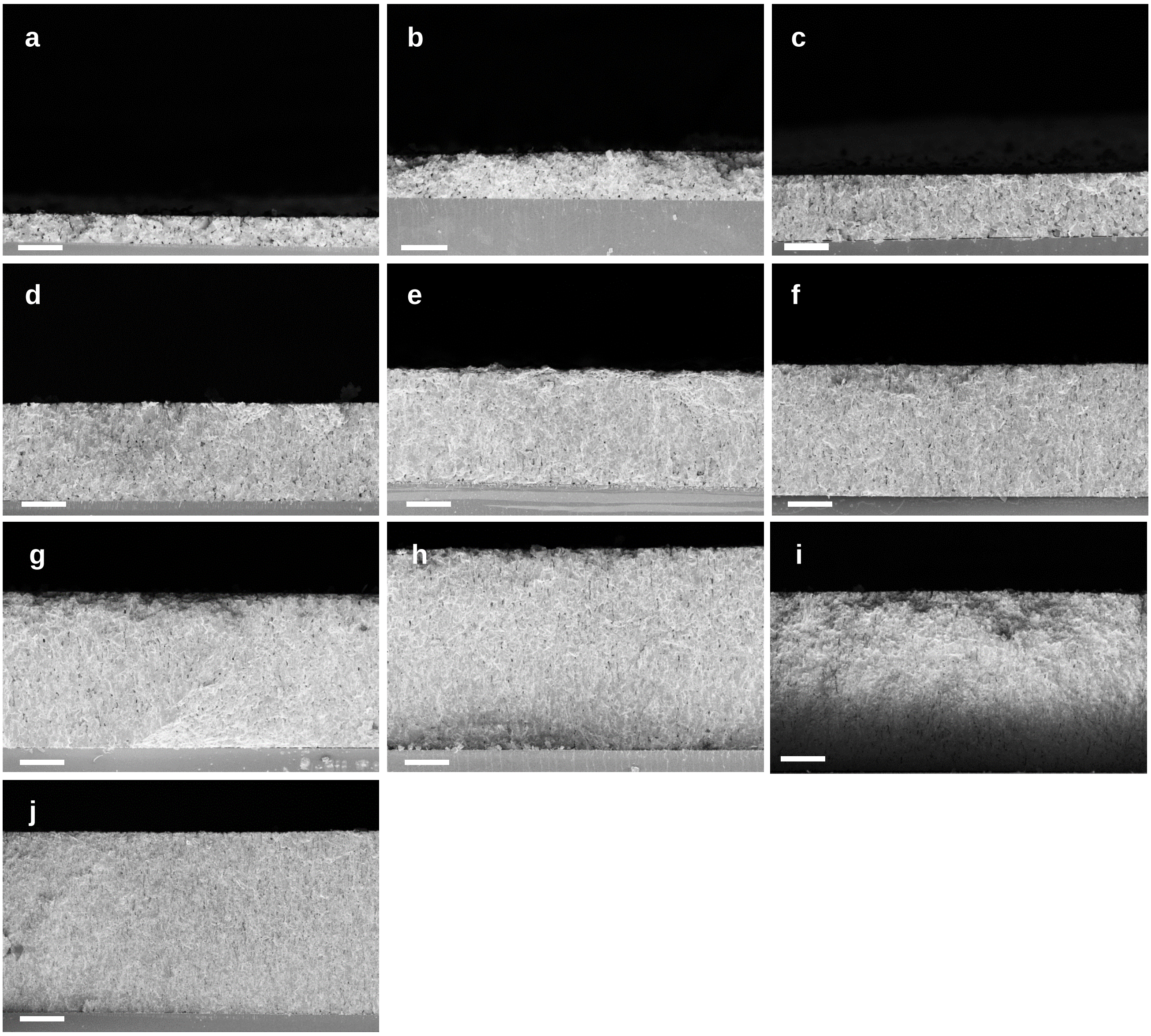


**Supplementary Fig. 9 | Thick $CsPbBr_3$ films obtained through screen-printing process.** The $CsPbBr_3$ films with thickness from 50 to 600 $\mu m$ were successfully printed by controlling the printing pressure, frequency and other parameters. **a**, 50 $\mu m$ thick, the scale bar denotes 100 $\mu m$. **b**, 100 $\mu m$ thick, the scale bar denotes 100 $\mu m$. **c**, 150 $\mu m$ thick, the scale bar denotes 100 $\mu m$. **d**, 200 $\mu m$ thick, the scale bar denotes 100 $\mu m$. **e**, 250 $\mu m$ thick, the scale bar denotes 100 $\mu m$. **f**, 300 $\mu m$ thick, the scale bar denotes 100 $\mu m$. **g**, 350 $\mu m$ thick, the scale bar denotes 100 $\mu m$. **h**, 400 $\mu m$ thick, the scale bar denotes 100 $\mu m$. **i**, 450 $\mu m$ thick, the scale bar denotes 200 $\mu m$. **j**, 500 $\mu m$ thick, the scale bar denotes 200 $\mu m$.

## The crystallization process of $CsPbBr_3$ thick film

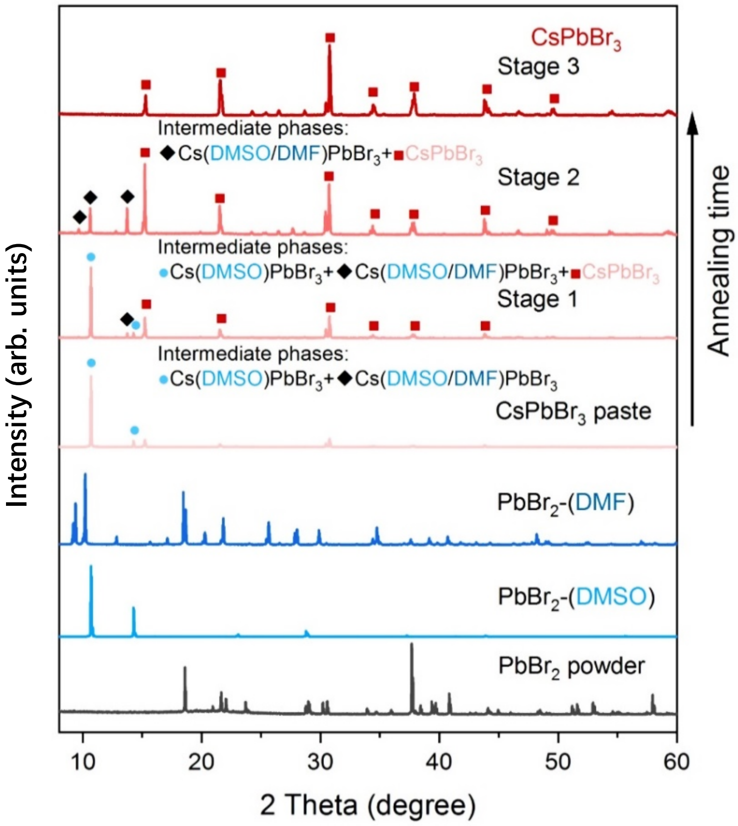


**Supplementary Fig. 10 | The crystallization of the $CsPbBr_3$ thick film during thermal annealing.** The $CsPbBr_3$ paste prepared with such binary solvents at a volume ratio of 7:3 (DMSO:DMF) presents the main intermediate phase of Cs(DMSO)$PbBr_3$. Under solvent evaporation, the nucleation and crystal growth of $CsPbBr_3$ can be observed from the increased diffraction signals of $CsPbBr_3$ at stage 1. Also, the diffraction signals belong to intermediate phase of Cs(DMSO/DMF)$PbBr_3$ appears. With further annealing, the main intermediate phase changes to Cs(DMSO/DMF)$PbBr_3$ at stage 2, suggesting that the thermal stability of Cs(DMSO/DMF)$PbBr_3$ is much better than that of Cs(DMSO)$PbBr_3$. Finally, the Cs(DMSO/DMF)$PbBr_3$ decomposes and $CsPbBr_3$ thick film forms at stage 3 under continuous annealing. Such gradual decomposition of intermediate phases may be responsible for the formation of high-quality $CsPbBr_3$ thick film.

## The fabrication of $CsPbBr_3$ thick film through screen-printing

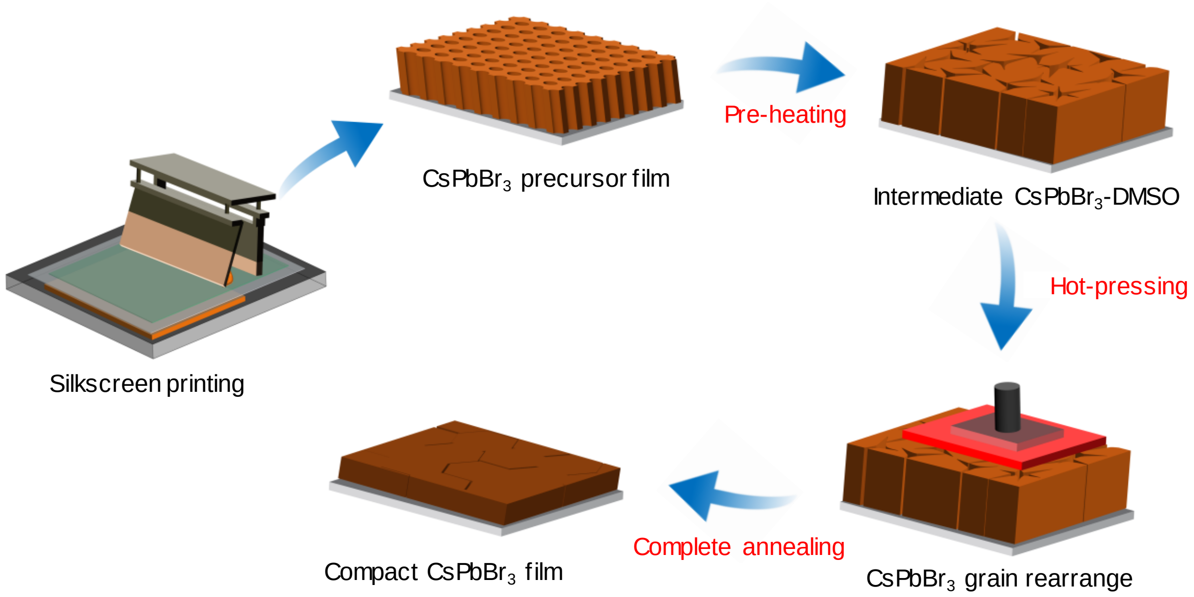


**Supplementary Fig. 11 | Schematic illustration of the fabrication of $CsPbBr_3$ thick film through screen-printing with hot-pressing treatment.** The screen-printed $CsPbBr_3$ precursor film was pre-heated at 100 °C for 10 min, obtaining an intermediate Cs(DMSO/DMF)$PbBr_3$ thick film. Afterwards, the intermediate film was hot-pressed at 150 °C and 0.5 MPa for 10 min inside a self-designed equipment. During this procedure, the perovskite grains were rearranged, resulting in a dense morphology. Finally, the film was heated at 150 °C for 30 min, obtaining a compact $CsPbBr_3$ thick film.

## The analysis results of the intermediate perovskite film

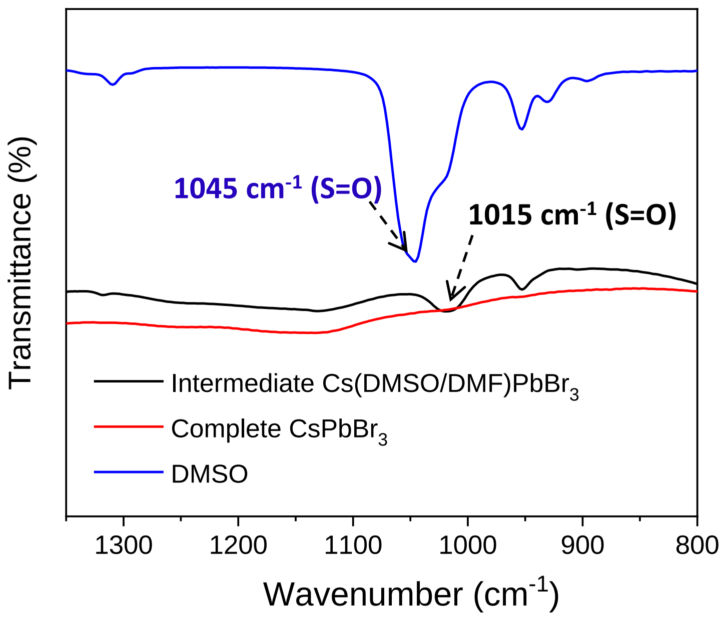


**Supplementary Fig. 12 | The Fourier transform infrared (FTIR) spectra of the intermediate Cs(DMSO/DMF)$PbBr_3$, complete $CsPbBr_3$ and DMSO.** The intermediate Cs(DMSO/DMF)$PbBr_3$ exhibits an additional peak at 1015 $cm^{-1}$ ascribed to the stretching mode of S=O in DMSO, and the characteristic S=O stretching peak of DMSO at 1045 $cm^{-1}$ is downshifted to 1015 $cm^{-1}$ for the intermediate Cs(DMSO/DMF)$PbBr_3$, which can be explained by the coordination between O and Pb.

## The hot-pressed $CsPbBr_3$ film

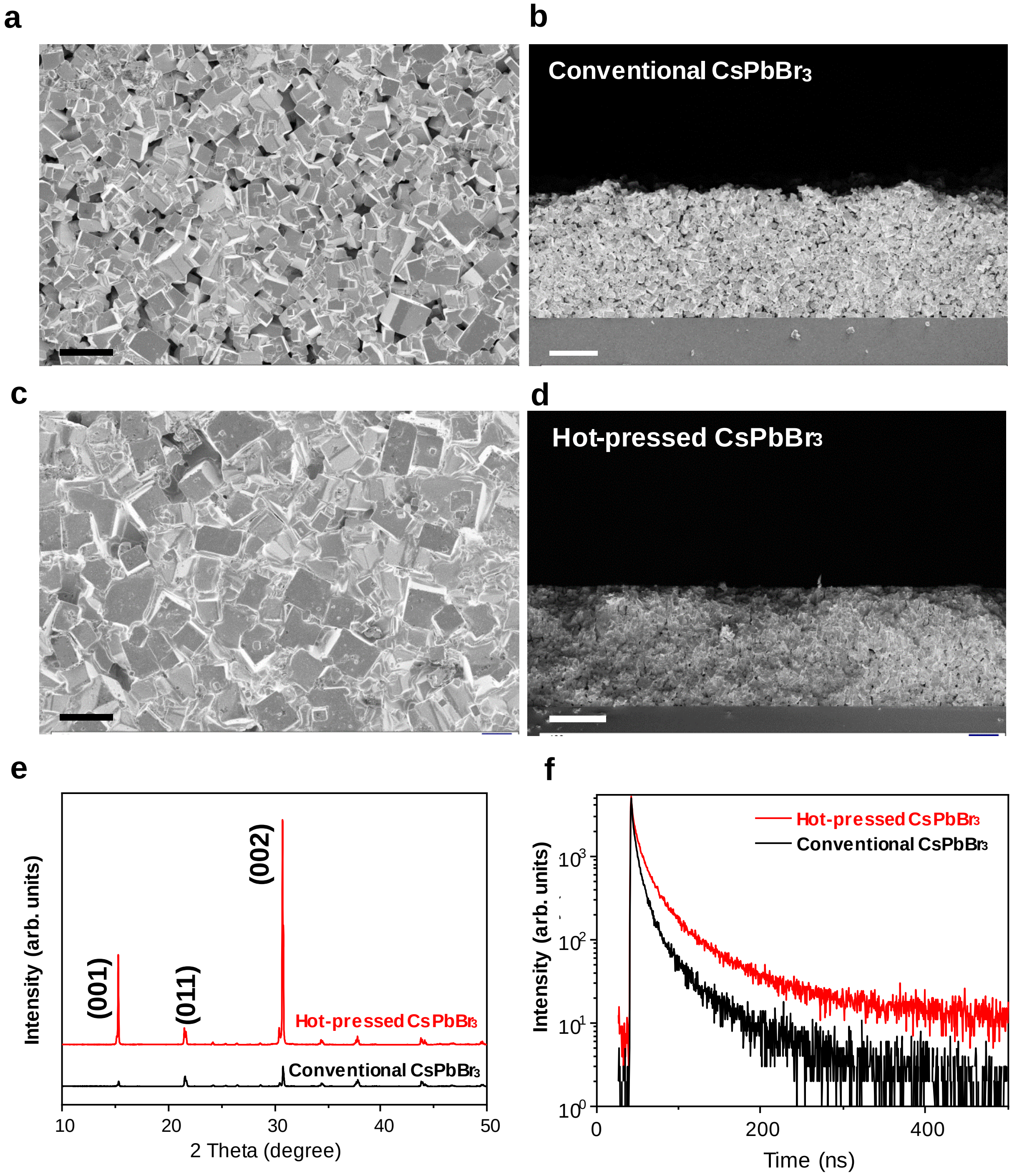


**Supplementary Fig. 13 | The scanning electron microscope (SEM) and characterization results of the hot-pressed $CsPbBr_3$ film. a,** top-view SEM image of the conventional $CsPbBr_3$ thick film. The scale bar denotes 30 μm. **b,** cross-section SEM image of the conventional $CsPbBr_3$ thick film. The scale bar denotes 100 μm. **c,** top-view SEM image of the hot-pressed $CsPbBr_3$ thick film. The scale bar denotes 30 μm. **d,** cross-section SEM image of the hot-pressed $CsPbBr_3$ thick film. The scale bar denotes 100 μm. **e,** XRD analysis results of the conventional and hot-pressed $CsPbBr_3$ thick film. The hot-pressed $CsPbBr_3$ thick film exhibits a significantly enhanced crystallinity. **f,** time resolved photoluminescence (PL) of the conventional and hot-pressed $CsPbBr_3$ thick film. The hot-pressed $CsPbBr_3$ thick film shows a longer PL life-time than the conventional one.

## The configuration of the X-ray detector

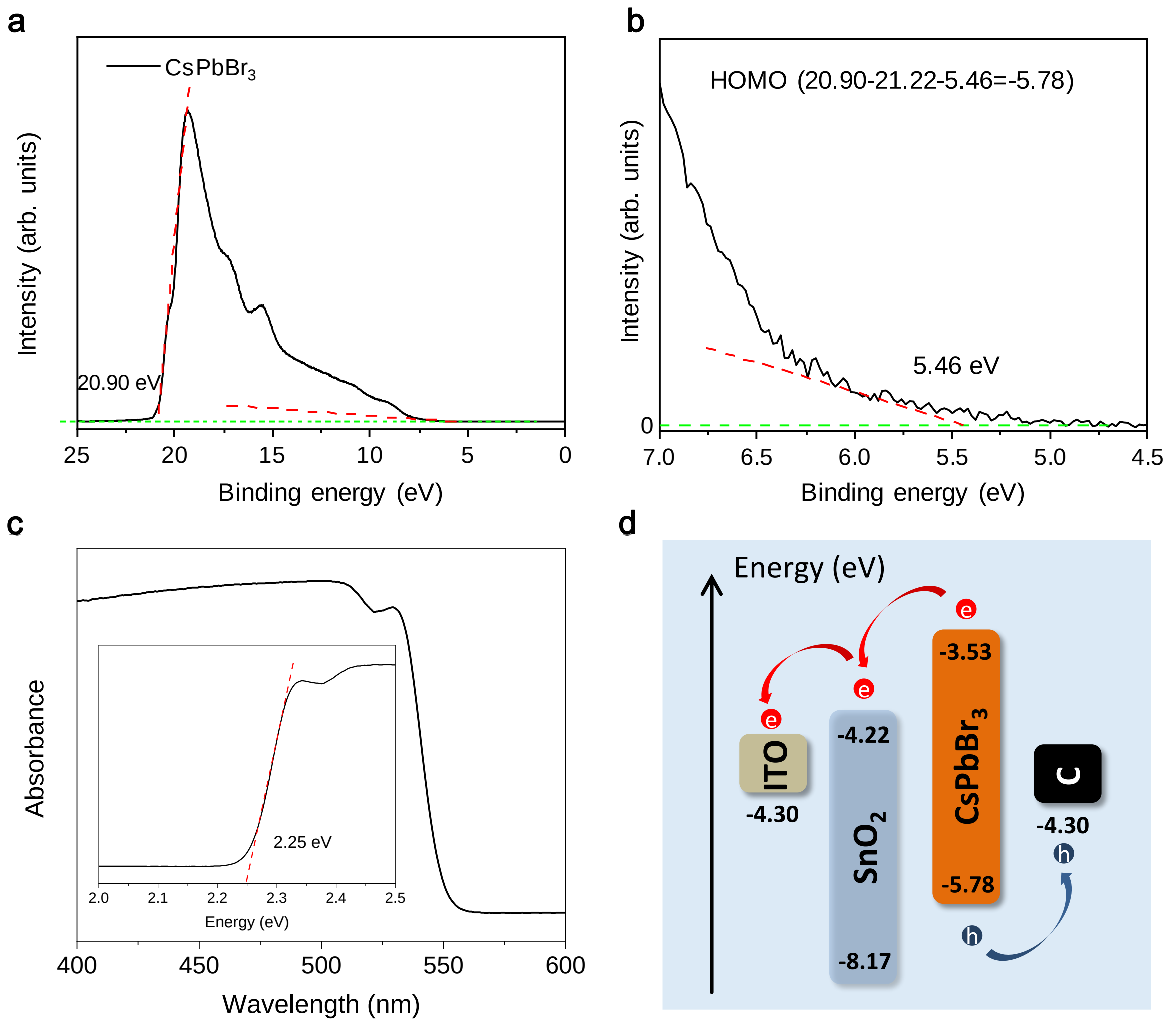


**Supplementary Fig. 14 | Configuration of the $CsPbBr_3$ X-ray detector. a,** Spectra of the $CsPbBr_3$ thick film measured from Ultraviolet photoelectron spectroscopy (UPS). The highest occupied molecular orbital (HOMO) of the $CsPbBr_3$ is calculated to be -5.78 eV. **b,** Zoomed-in UPS spectra of the $CsPbBr_3$ thick film with binding energy between 4.5 eV to 7.0 eV. **c,** UV-visible absorption of the $CsPbBr_3$ film. The bandgap of $CsPbBr_3$ film is 2.25 eV. **d,** The energy level diagram of the $CsPbBr_3$ X-ray detector.

## The photocurrent responses of the X-ray detector at various electric fields

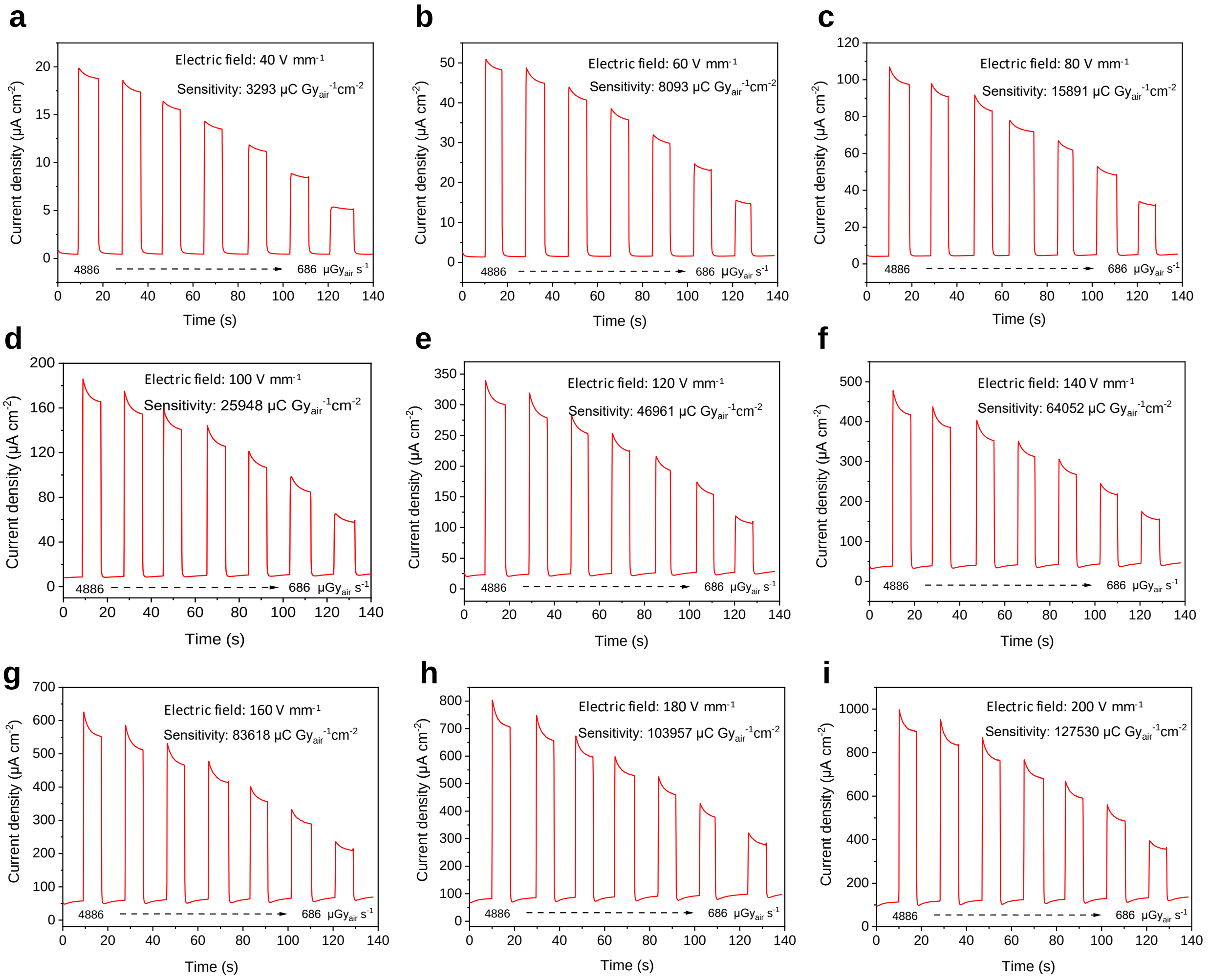


**Supplementary Fig. 15 | The X-ray detector photocurrent responses under different X-ray dose rates at various electric fields.** The X-ray detector exhibits a strong linear photocurrent response to the X-ray dose rates at various electric fields. However, high electric fields (⩾ 200 V mm$^{-1}$) may result in tremendous increase of noise current. **a**, the electric field is 40 V mm$^{-1}$. **b**, the electric field is 60 V mm$^{-1}$. **c**, the electric field is 80 V mm$^{-1}$. **d**, the electric field is 100 V mm$^{-1}$. **e**, the electric field is 120 V mm$^{-1}$. **f**, the electric field is 140 V mm$^{-1}$. **g**, the electric field is 160 V mm$^{-1}$. **h**, the electric field is 180 V mm$^{-1}$. **i**, the electric field is 200 V mm$^{-1}$.

## The photocurrent responses of the detector at high electric fields

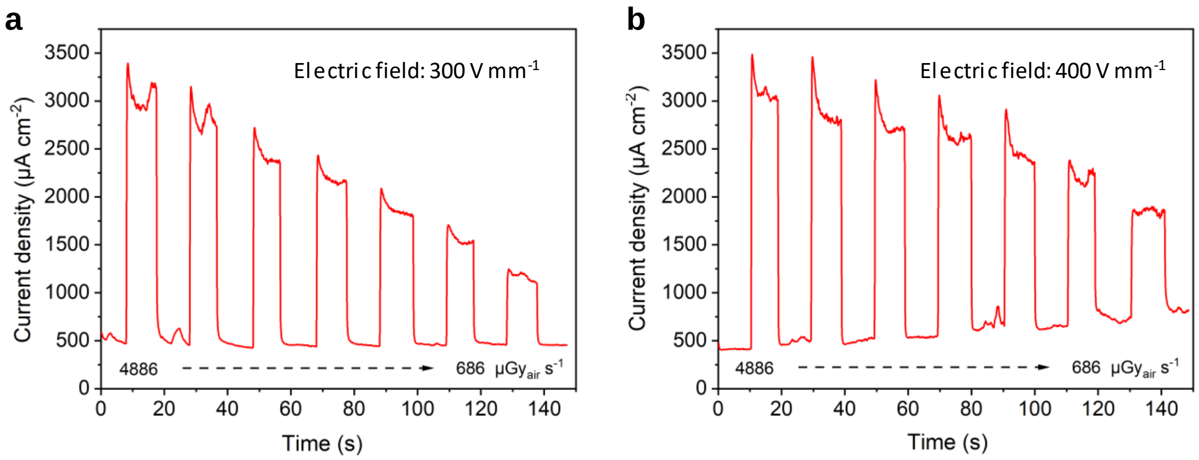


**Supplementary Fig. 16 | The X-ray detector photocurrent responses at high electric fields.** The linear photocurrent responses start to vanish at high electric field. The X-ray detector tended to break down at high bias voltages with distorted response current. **a**, the electric field is 300 V mm$^{-1}$. **b**, the electric field is 400 V mm$^{-1}$.

## The lower limit-of-detection results of the X-ray detector

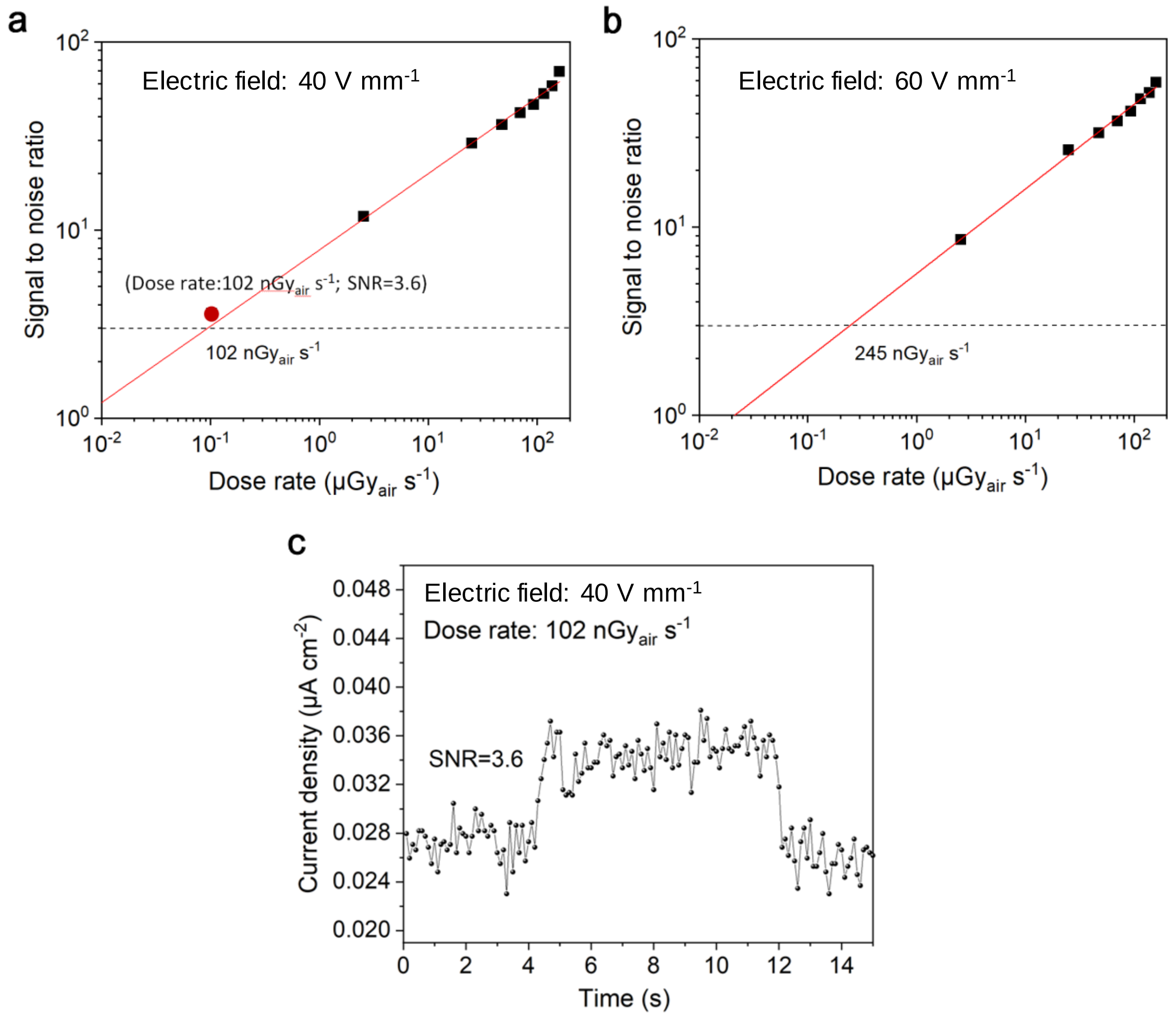


**Supplementary Fig. 17 | The lower limit-of-detection (LoD) responses of the X-ray detector.** **a**, the LoD of $CsPbBr_3$ detector was 102 $nGy_{air}$ $s^{-1}$ at electric field of 40 V $mm^{-1}$. The red dot is obtained from experimental measurements of current density under electric field of 40 V $mm^{-1}$ and dose rate of 102 $nGy_{air}$ $s^{-1}$. **b,** the LoD of $CsPbBr_3$ detector was 245 $nGy_{air}$ $s^{-1}$ at electric field of 60 V $mm^{-1}$. **c**, statistically, the measured photocurrent was 3.6 times higher than the dark noise when the dose rate was 102 $nGy_{air}$ $s^{-1}$.

## The cycling tests of the detector with X-ray on and off

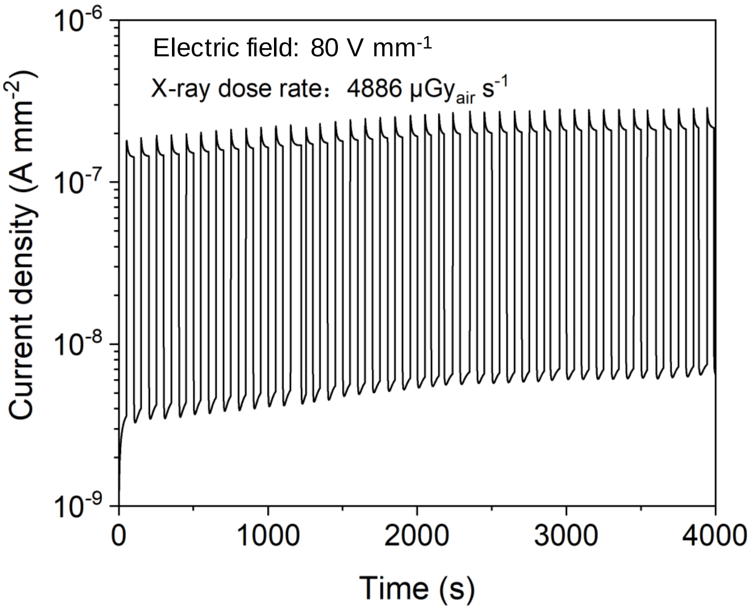


**Supplementary Fig. 18 | The cycling tests of the detector with X-ray on and off for more than one hour.** The $CsPbBr_3$ X-ray detector exhibits a satisfactory working stability with low current drift.

## X-ray diffraction and photoluminescence spectroscopy analysis results

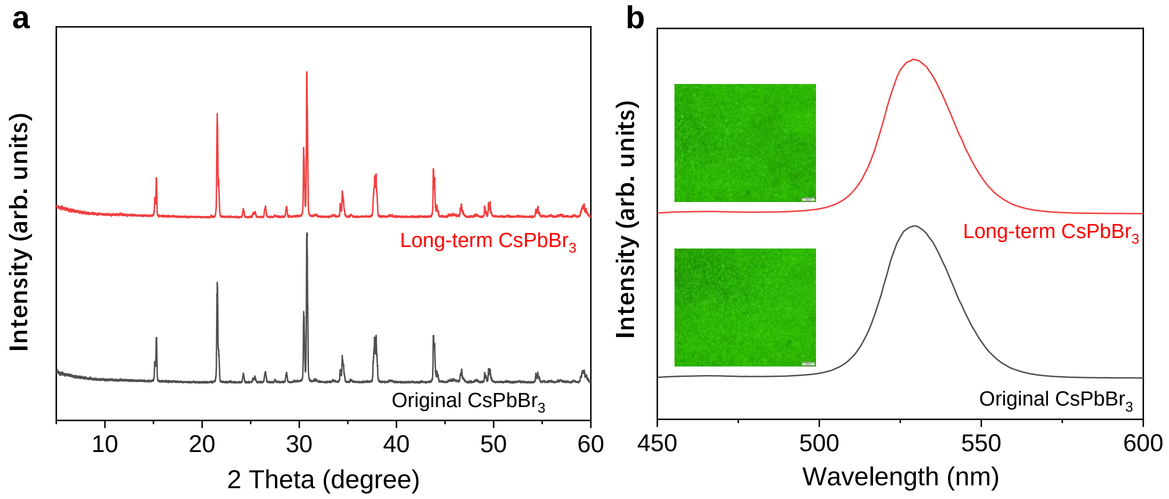


**Supplementary Fig. 19 | The X-ray diffraction (XRD) and photoluminescence (PL) spectroscopy analysis results of the original and long-term tested $CsPbBr_3$ film. a,** the long-term tested $CsPbBr_3$ thick film exhibits comparable XRD pattern with the original $CsPbBr_3$ thick film, that indicates the crystal structure of the $CsPbBr_3$ perovskites is stable under constant X-ray irradiation. **b,** the $CsPbBr_3$ perovskites remains uniform and strong green luminescence after long-term irradiation. Above results indicated a material irradiation stability of our $CsPbBr_3$ thick film.

## The configuration of the imaging system

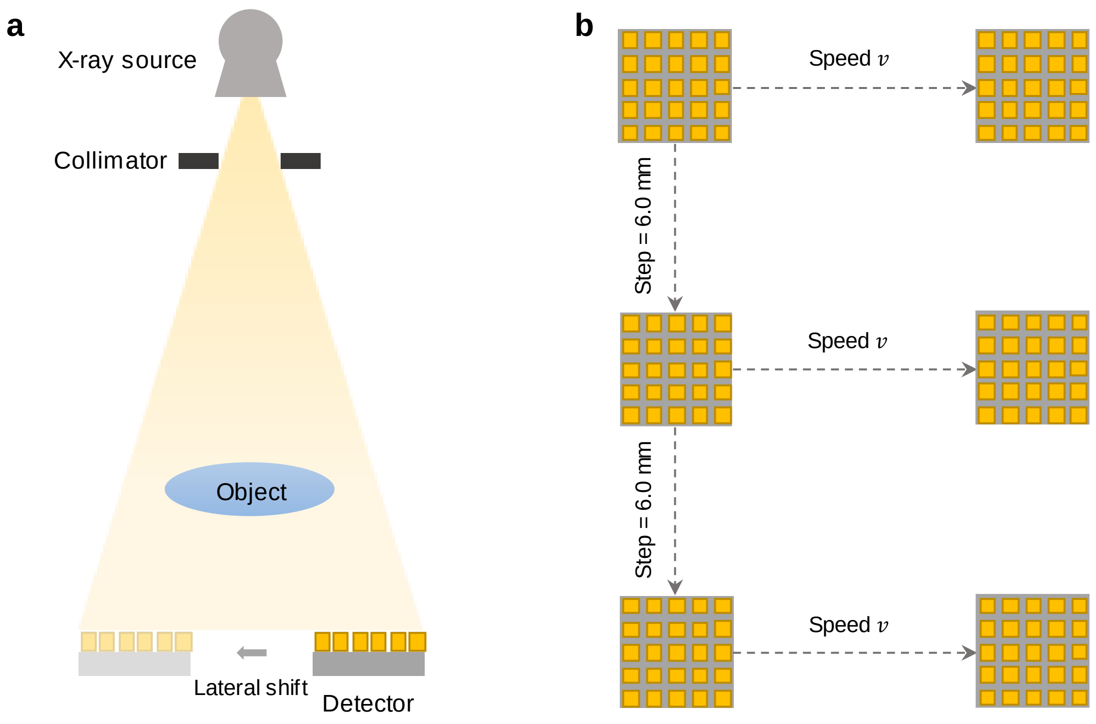


**Supplementary Fig. 20 | Illustrations of the imaging configuration. a,** the X-ray imaging settings. The source to object distance is 440 mm, and the source to detector distance is 476 mm. The CMOS detector scans the object laterally with a certain speed. **b,** illustration of the 2D scanning of the CMOS detector along the vertical and horizontal directions. Specifically, the CMOS detector steps consecutively along the vertical direction with a 6.0 mm step size.

## The experimental setup

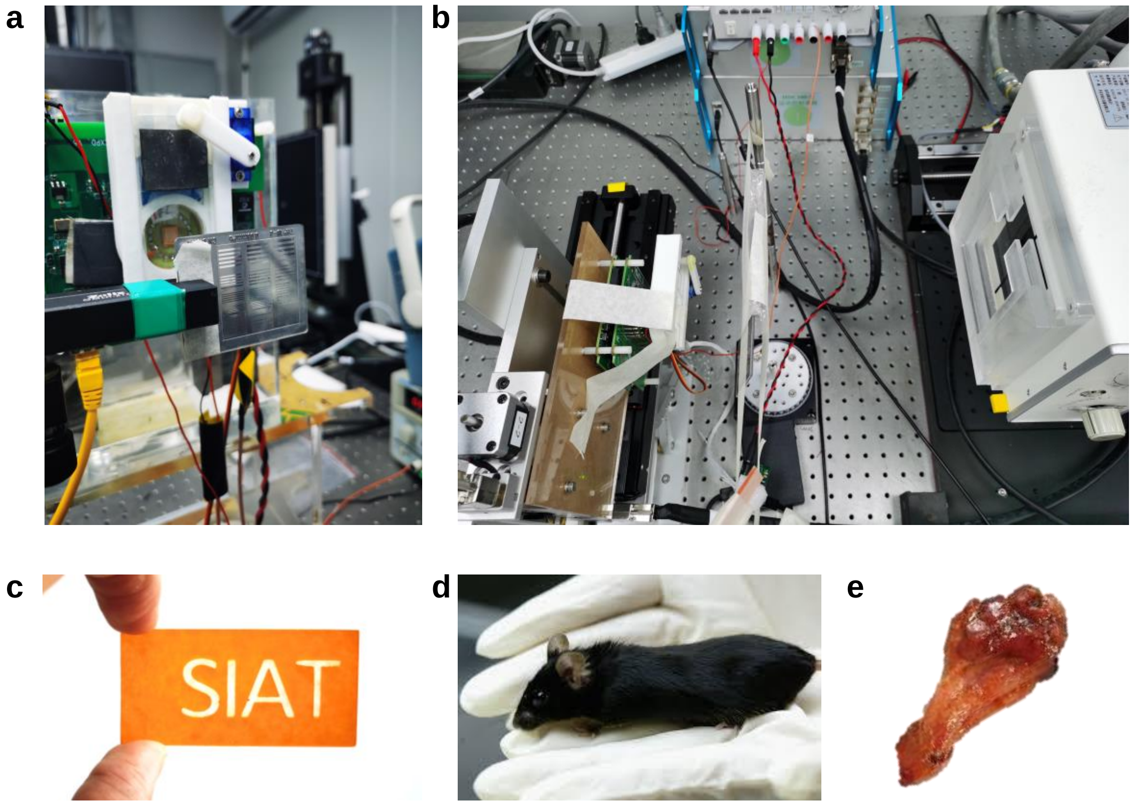


**Supplementary Fig. 21 | Experimental system and some imaging samples. a,** the experimental setup for the resolution bar pattern. **b,** the experimental setup for the mouse specimen. **c,** the 3D printed sample (thickness of 2.0 mm). **d,** the mouse specimen. **e,** the chicken drumette specimen.

## The detector readout circuit

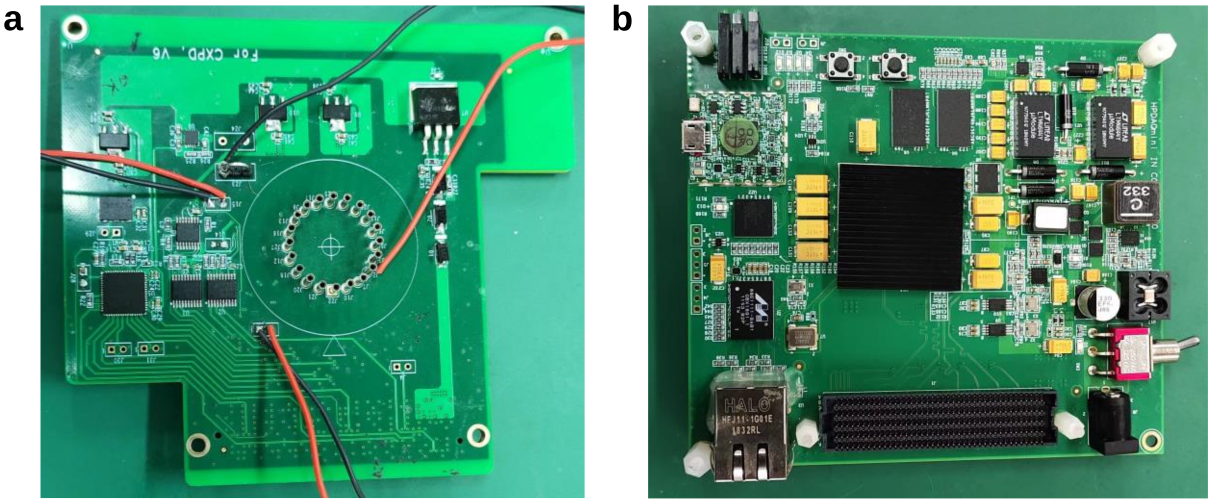

**Supplementary Fig. 22 | The readout circuits of this CMOS X-ray detector. a,** mixed signal converter board. **b,** Field-Programmable Gate Array (FPGA) based control board. Online signals are transferred to the workstation via a standard Ethernet cable.

# The optimal bias voltage

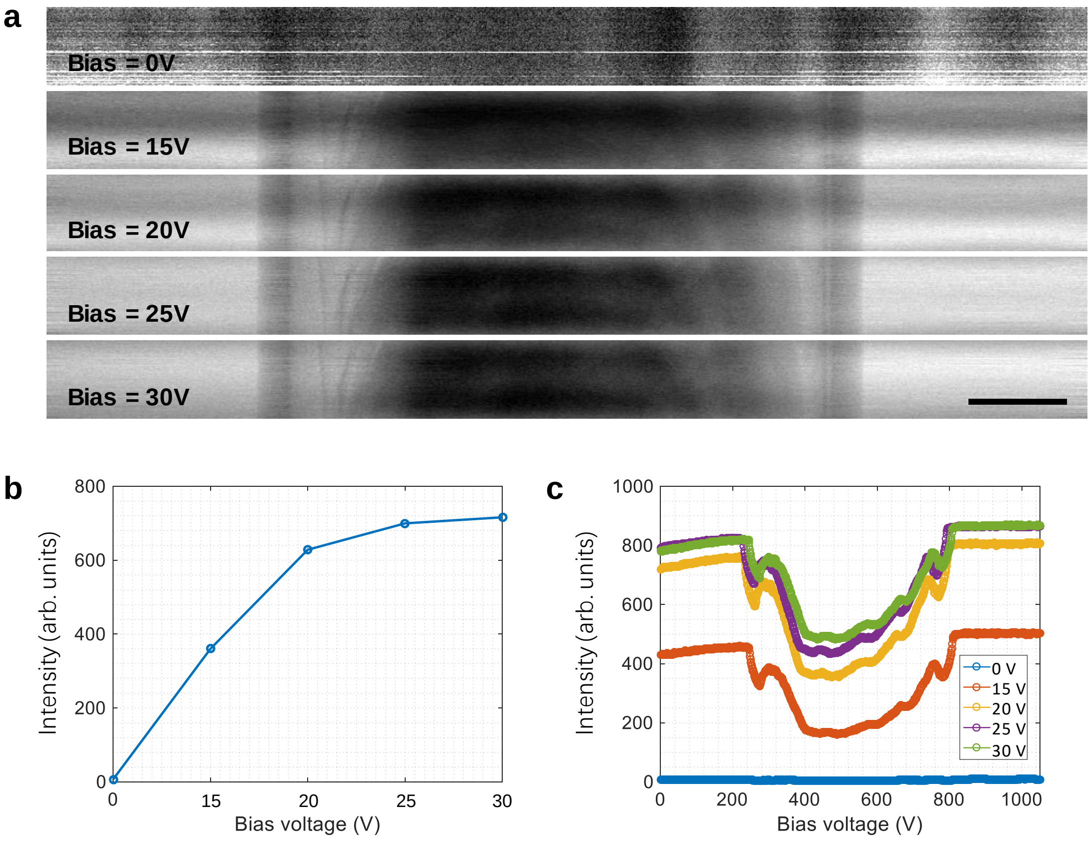


**Supplementary Fig. 23 | CMOS detector responses at varied bias voltages. a,** acquired DR images of a sample at five different bias voltages (0, 15, 20, 25 and 30 V), respectively. At 0 V, the detector has very tiny response, and the object can be hardly recognized. Images are displayed with self-adjusted window and level. The scale bar denotes 5.0 mm. **b,** overall responses of the CMOS detector at different bias voltages. As can be seen, the detector response increases as the bias voltage becomes higher. At 25 V and 30 V, the detector responses get saturated. As a result, we selected 25 V as the bias voltage. **c,** line profile (vertically averaged signal) comparison results.

## The detector responses and corrections

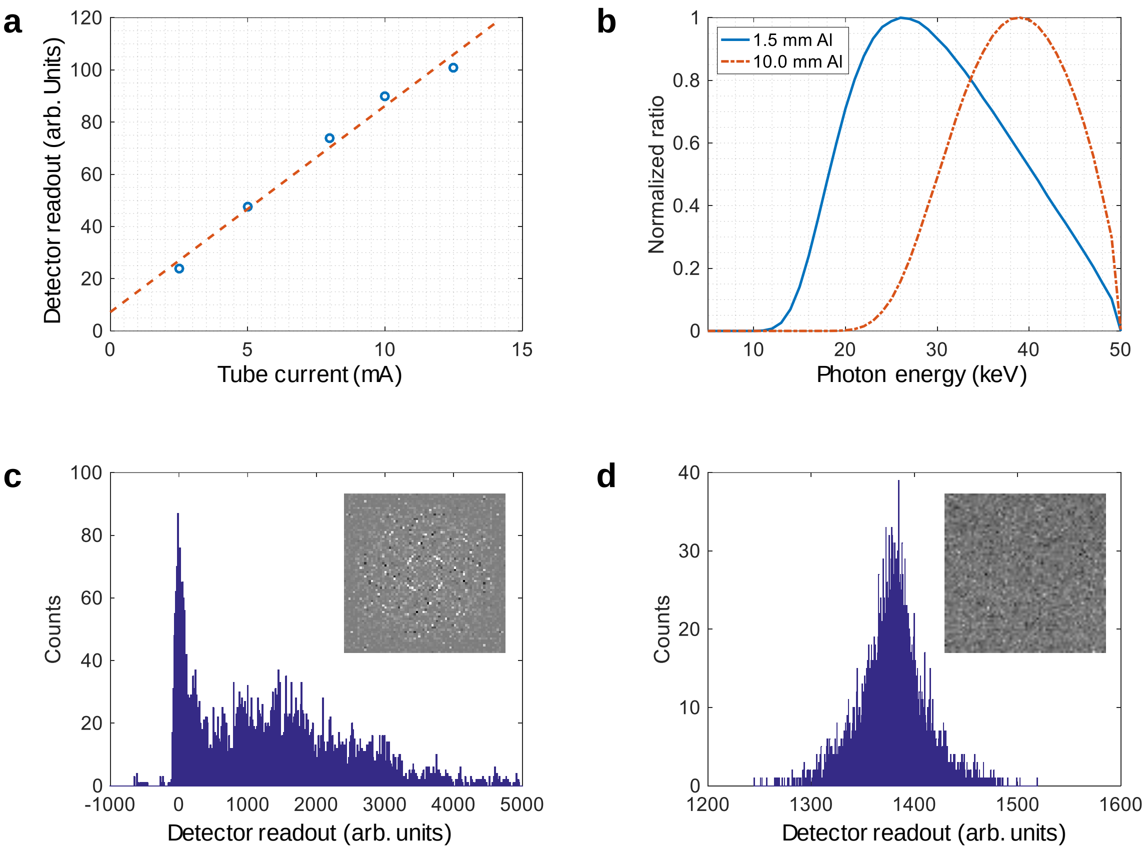


**Supplementary Fig. 24 | Detector response and image correction results. a,** responses of the CMOS X-ray detector under different tube current values (2.5, 5.0, 8.0, 10.0 and 12.5 mA). These measurements show good linearity of the detector response. **b,** beam spectra used in this study. In particular, the 10.0 mm Al filtered beam is used as the RQA3 beam to measure the MTF and NPS, and the 1.5 mm Al filtered beam is used to measure the specimen. **c,** raw image and distribution histogram before gain correction. **d,** raw image and distribution histogram after gain correction. Image pixels get uniform values after the gain correction.

## The temporal response current of the detector to square-wave X-ray

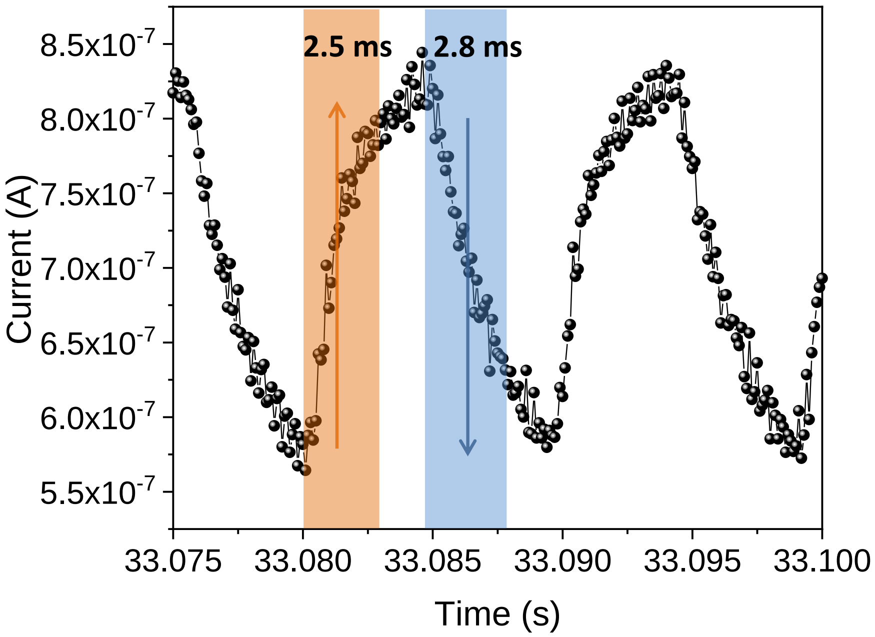


**Supplementary Fig. 25 | The temporal response of the X-ray detector to square-wave X-rays.** The temporal response current of the $CsPbBr_3$ X-ray detector to square-wave X-rays is very sharp with a rising-time of 2.5 ms and a falling-time of 2.8 ms.

## The modulation transfer function analysis

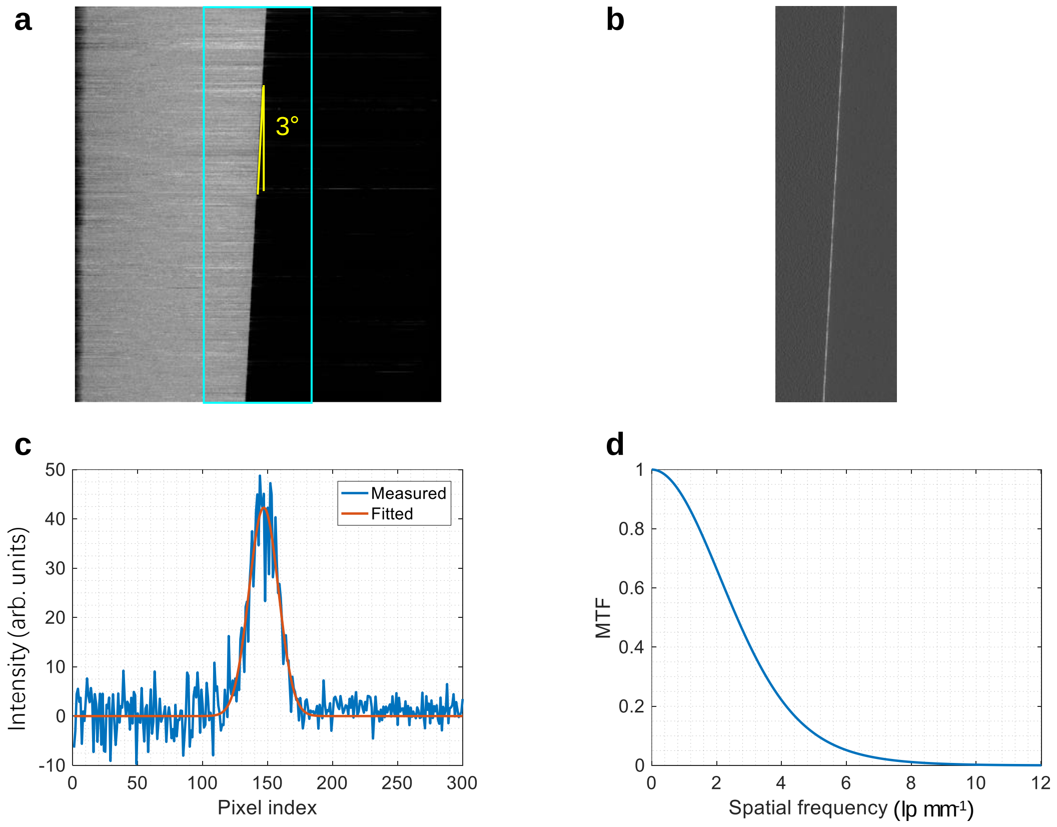


**Supplementary Fig. 26 | Modulation transfer function (MTF) analysis results. a,** acquired X-ray image of the tungsten plate (tilted by 3 degrees with respect to the vertical direction), corresponding to the edge spread function. **b,** calculated line spread image from the edge spread image. **c,** measured pixel intensity distribution profile (blue line), and the Gaussian function fitted line spread function (LSF, orange line). **d,** calculated MTF curve after performing the Fast Fourier Transform (FFT) on the LSF.

## The noise power spectrum analysis

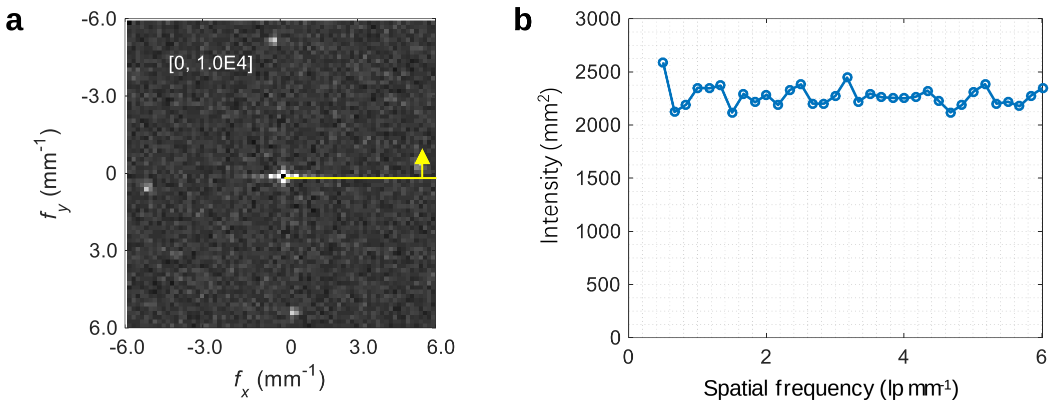


**Supplementary Fig. 27 | Noise power spectrum (NPS) analysis results. a,** calculated 2D distributed NPS map of the CMOS detector. The bright spots are caused by the internal fixed structures on CMOS. Usually, these spots can be ignored. **b,** 1D NPS curve after radially averaging the 2D NPS map, as indicated by the yellow line and arrow on the 2D NPS map. The almost flatten profile demonstrates that this CMOS detector exhibited a white noise.

## The static X-ray imaging results

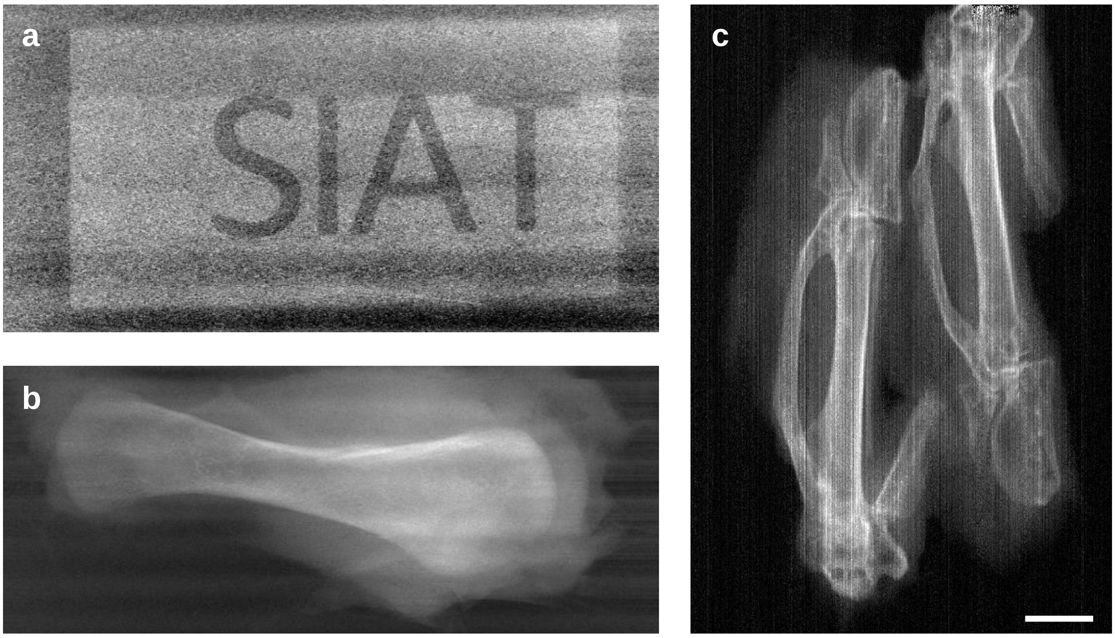


**Supplementary Fig. 28 | Additional X-ray imaging results obtained from this CMOS perovskite X-ray detector. a,** DR image of the 3D printed plastic plate with SIAT logo. **b,** DR image of a chicken drumette specimen. **c,** DR image of a chicken wing specimen. The scale bar denotes 10 mm.

## The dynamic X-ray imaging results

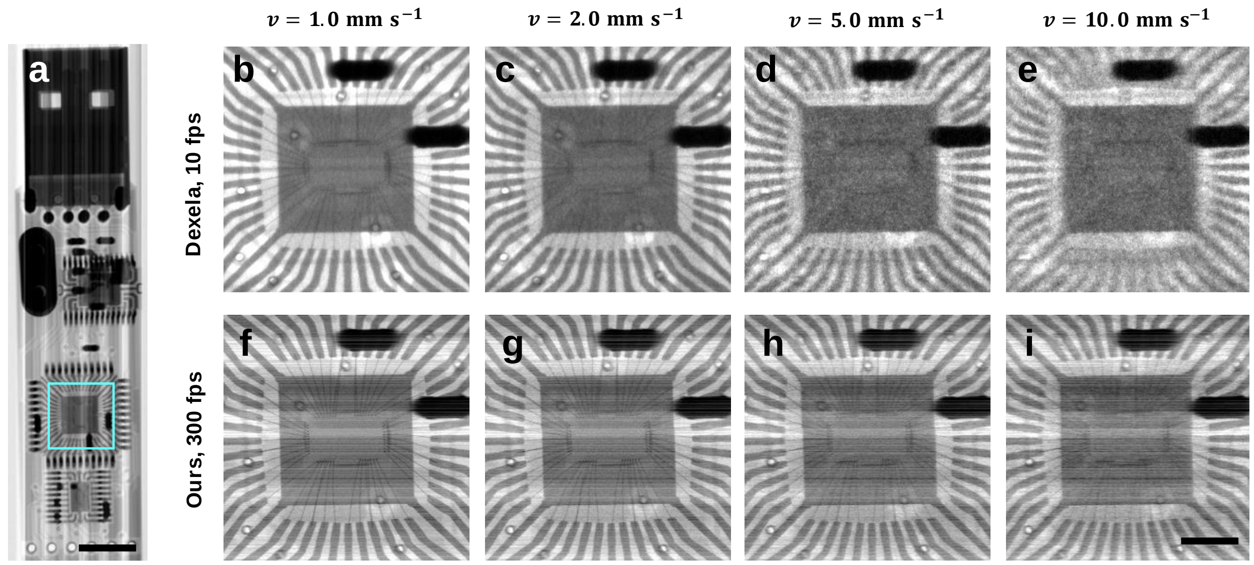


**Supplementary Fig. 29 | The dynamic X-ray imaging results of a USB flash drive obtained at different scanning speeds. a,** the X-ray imaging result of a USB flash drive. The scale bar denotes 10.0 mm. **b,** the zoomed-in Dexela result obtained at 1.0 mm $s^{-1}$. **c,** the zoomed-in Dexela result obtained at 2.0 mm $s^{-1}$. **d,** the zoomed-in Dexela result obtained at 5.0 mm $s^{-1}$. **e,** the zoomed-in Dexela result obtained at 10.0 mm $s^{-1}$. **f,** the zoomed-in Pervoskite result obtained at 1.0 mm $s^{-1}$. **g,** the zoomed-in Pervoskite result obtained at 2.0 mm $s^{-1}$. **h,** the zoomed-in Pervoskite result obtained at 5.0 mm $s^{-1}$. **i,** the zoomed-in Pervoskite result obtained at 10.0 mm $s^{-1}$. The scale bar denotes 2.0 mm. As seen, the new perovskite CMOS X-ray detector with fast readout speed provides superior image quality than the commercial Dexela detector, particularly when the object is scanned at higher speeds.
